# Objective lameness assessment of 235 horses undergoing lameness examination in Brazil: A retrospective study

**DOI:** 10.29374/2527-2179.bjvm008224

**Published:** 2025-05-22

**Authors:** Ana Paula da Costa Rodrigues, Ricardo Pozzobon, Grasiela De Bastiani, Flávio Desessards De La Côrte, Marcos da Silva Azevedo

**Affiliations:** 1 Programa de Pós-Graduação em Ciência Animal, Setor de Clínica e Cirurgia de Grandes Animais, UNIPAMPA, Uruguaiana, RS, Brazil.; 2 Departamento de Clínicas de Grandes Animais, UFSM, Santa Maria, RS, Brazil.; 3 Faculdade de Medicina Veterinária, Universidade Federal do Rio Grande do Sul, Porto Alegre, RS, Brazil.; 4 Setor de Clínica e Cirurgia de Grandes animais, UNIPAMPA, Uruguaiana, RS, Brazil.

**Keywords:** epidemiology, horse, lameness locator, orthopedic, wireless inertial sensor, epidemiologia, cavalo, localizador de claudicação, ortopédico, sensor inercial sem fio

## Abstract

Lameness, which arises from functional or structural changes in the limbs or axial skeleton, causes asymmetry in the movement of the equine head and/or pelvis. This study aimed to investigate the lameness patterns of horses that underwent lameness examination or monitoring during the years 2016 to 2020. This retrospective study used data from the lameness examination, evaluated using an objective assessment with body-mounted wireless inertial sensors. The lameness examination comprised clinical history, static inspection, palpation, gait evaluation (for which the animals were equipped with a wireless inertial sensor system), flexion tests, lunging examination, diagnostic anesthetic blocks, and imaging examinations. Based on objective assessment data, the condition of lameness, limb with primary lameness, type of lameness, intensity, and location of lameness were determined. Of the 235 animals included in this study, 93,6% presented lameness. Of the animals with lameness, 59,5% had forelimb lameness and 40,5% had hindlimb lameness. The most frequent lameness condition was primary lameness in one limb and secondary lameness in the other. Impact lameness was the most frequent type in both the forelimb and hindlimb. Moderate-to-severe lameness was the most frequent level of intensity. Regarding the lameness location, in the forelimb, the distal region was the most affected, while in the hindlimb, the proximal lower region was the most affected. We conclude that forelimb lameness is more frequent in horses examined in southern Brazil and mainly affects the distal limb region.

## Resumo

A claudicação, decorrente de alterações funcionais ou estruturais dos membros ou do esqueleto axial, causa assimetria no movimento da cabeça e/ou pelve equina. O objetivo deste estudo foi investigar os padrões de claudicação de um grupo de cavalos apresentados para exame ou monitoramento de claudicação, durante os anos de 2016 a 2020. Este estudo retrospectivo utilizou dados do exame de claudicação, avaliados por meio de avaliação objetiva com sensores inerciais sem fio montados no corpo. O exame de claudicação consistiu em história clínica, inspeção estática, palpação, avaliação da marcha (para a qual os animais foram equipados com um sistema de sensor inercial sem fio), testes de flexão, exame de avanço, bloqueios anestésicos diagnósticos e exames de imagem. Com base nos dados da avaliação objetiva, foram determinados a condição de claudicação, o membro com claudicação primária, o tipo de claudicação, a intensidade e a localização da claudicação. Dos 235 animais incluídos no estudo, 93,6% apresentaram claudicação. Dos casos de claudicação, 59,5% apresentaram claudicação de membro anterior e 40,5% de membro posterior. De acordo com a condição de claudicação, a mais frequente foi a claudicação primária em um membro e secundária no outro. A claudicação de impacto foi o tipo mais frequente, tanto no membro anterior quanto no membro posterior. A claudicação moderada a grave foi o nível de intensidade mais frequente. Quanto à localização da claudicação, no membro anterior, a região distal foi a mais acometida, e no membro posterior, a região proximal inferior foi a mais acometida. Concluímos que a claudicação de membro anterior é mais frequente em equinos examinados no sul do Brasil, acometendo principalmente a região distal do membro.

## Introduction

Lameness is evidenced by a left- or right-sided asymmetry during motion, which is usually reflected in the movement of the equine head and pelvis (Baxter et al., 2020). Lameness is one of the most common conditions in horses, causing huge economic losses to owners (Seitzinger et al., 2000), and is considered the most common problem in horses in the United Kingdom (Murray et al., 2010). In addition, many horses must be removed from competition, as reported in a study in which 57.8% of the animals were eliminated from competition because of lameness (Nagy et al., 2017). In Brazil, lameness is one of the most common conditions in athletic horses, with a prevalence ranging from 47.6% (Menarim et al., 2012) to 75% (Moreira et al., 2019). Mora-Carreno et al. (2014) identified spontaneous lameness in 98.3% of Chilean rodeo horses. Rossdale et al. (1985) reported that training had to be interrupted in 67.6% of the animals because of lameness problems, and Baccarin et al. (2012) identified lameness in 78% of animals with osteoarthritis.

Subjective assessment is an accepted practical standard for the detection and clinical quantification of lameness (Baxter et al., 2020). However, subjective assessment of lameness in horses has shown low interobserver and intraobserver agreement (Keegan, 2007). In addition, there may be a hasty evaluation by clinicians regarding the expected or desired results after anesthetic blocks are administered (Schumacher et al., 2014). Therefore, the use of objective methods to detect and quantify lameness in both clinical and research settings has increased (Keegan, 2007; Schumacher et al. 2014).

Because lameness is one of the main disorders affecting horses, more studies need to be conducted to help veterinarians understand this condition. Thus, the aim of this study was to report the type and lameness distribution quantified using a sensor-based gait analysis system and compare it to a clinical investigation.

## Materials and methods

### Animals

This retrospective study used data from 2016 to 2020 from lameness examinations of horses in the southern region of Brazil that were performed by our team. This was done by assessing data from the horses with an objective assessment system based on wireless inertial sensors (Equinosis Q with Lameness Locator®). The inclusion criteria for this study included the need for a complete evaluation of lameness using objective assessment, as well as identification of the lameness location by diagnostic blocks or imaging exams. The animals included in this study were from different locations. The information recorded for each animal consisted of breed, equestrian activity, age, sex, and the type of surface on which the animal was examined during dynamic evaluation.

### Lameness examination

Evaluation of the locomotor system consisted of anamnesis, static inspection, palpation, examination while pacing and trotting in a straight line or in a lunge in both directions, manipulative tests, diagnostic anesthetic blocks, and imaging examinations. The palpation examination initially consisted of evaluating the hoof with hoof tongs, followed by palpation of the other limb structures in a distal-to-proximal direction. The animals were then evaluated, first walking in a straight line and in a figure-eight motion and then trotting in a straight line for approximately 20–30 m, both ways.

Subsequently, they were subjected to manipulative tests according to the clinician's choice and based on the findings from palpation and examination in motion. The main tests performed for the forelimbs were fetlock flexion, distal flexion, carpus flexion, and toe elevated test, whereas for the hindlimbs, the main tests were stifle flexion, distal flexion, and full limb flexion. The lunge examination in both directions was performed after the manipulative tests and only when the clinician deemed it necessary to exacerbate subtle lameness or assist in differentiating between lameness with a possible proximal or distal origin. The choice of the type of surface used for the animals' evaluation was based on availability at the sites, always prioritizing a more homogeneous type of floor. The physical examinations and manipulative tests were performed by the same clinician.

Anesthetic blocks were divided into perineural and intra-articular blocks, and their choice was based on clinical suspicion and findings. Perineural blocks were applied from the distal to the proximal limb region, starting with a palmar digital block in the forelimbs and an abaxial sesamoid block in the hindlimbs. Anesthetic blocks were applied as proposed by Moyer and Carter (2007), and the anesthetic of choice was lidocaine 2% and antisepsis with povidone-iodine, followed by cleaning with alcohol 70%. After each block, the animals were objectively evaluated after 5- and 10-min. Animals that showed an improvement equal to or greater than 60% after the anesthetic blocks were considered to have a positive response. Ultrasound and radiographic imaging were performed according to the results found during the clinical evaluation and the clients' financial availability, using the already known standard protocols for each region to be evaluated.

### Objective assessment of lameness

After the physical examination, the animals were equipped with a body-mounted system (Equinosis Q with Lameness Locator^®^) for objective assessment. This system was chosen for its ease of use, availability in the region where the study was conducted, and objective assessment that does not rely on the subjective analysis of a veterinarian, which may be subject to bias owing to the subjective nature of the method. This was performed during the trotting examination, manipulative tests, lunge evaluation, and diagnostic blocks. Thus, the horses were equipped with three wireless inertial sensors, two of which were accelerometers, one placed on the head (midline, slightly behind the ears) and one on the pelvis (at the highest level, midline between the sacral tubers), and a gyroscope placed on the right forelimb (dorsal midline of the phalanges). Subsequently, they were evaluated while moving at a trot in a straight line for 20–30 m to obtain at least 25 strides.

Next, the manipulative tests were performed, and lunge evaluations were performed when necessary. Then, another evaluation while moving at a straight trot was performed, as in the previous one, to compare the baseline and after the diagnostic blocks. After each evaluation, the generated data was used to quantify and qualify the asymmetry using graphs for the fore and hind limbs. The data generated included DiffMax (difference in maximum head height between the right and left forelimbs) and DiffMin of the head (difference in minimum head height between the right and left forelimbs). An animal with at least one DiffMax or DiffMin value greater than 6 mm and a standard deviation lower than the mean was considered asymmetric. For the forelimbs, it is possible to evaluate the vector sum (lameness intensity) value, which consists of the vector sum of the DiffMax and DiffMin values of the head and must be greater than 8.5 mm to be considered asymmetric (Keegan et al., 2004; Kramer et al., 2004; Keegan et al., 2011; McCracken et al., 2012).

Regarding hindlimb evaluation, either the pelvic DiffMax (difference in maximum height of the pelvis between the right and left hindlimbs) or pelvic DiffMin (difference in minimum height of the pelvis between the right and left hindlimbs) must be greater than 3 mm, with a standard deviation lower than the mean (Keegan et al., 2004; Kramer et al., 2004; Keegan et al., 2011; McCracken et al., 2012). Considering the data, we will have the lameness intensity and phase of the stride, in addition to the possible primary origin of the lameness.

### Categories

Based on the data from the objective assessment, it was possible to determine some categories of lameness patterns in the animals, including lameness condition, limb with primary lameness, lameness type, lameness intensity, and lameness location.

### Lameness condition

**Figure 1:**
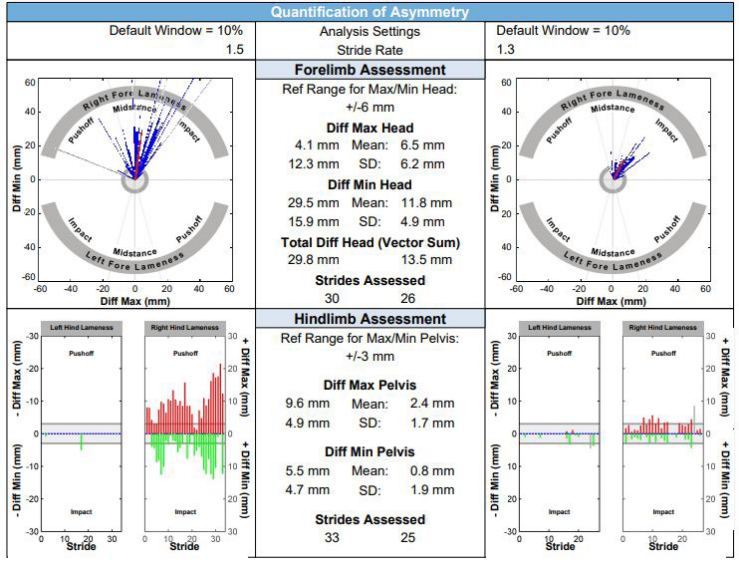
These graphs represent one of the animals included in the study after comparing two assessments: the left one before the block and the right one after the right stifle block. The yellow circle represents right hindlimb primary lameness, which improved by 100% after blocking. The black circle represents the compensatory lameness in the right forelimb, which significantly improved after blocking. The brown circle represents secondary lameness on the right forelimb, which remained after blocking.

The lameness condition was defined as: no lameness; primary lameness (a single limb with lameness, the limb with greater lameness intensity when more than one limb had lameness, or the limb for which diagnostic analgesia was first pursued) (Johnson et al., 2021); secondary lameness (the less lame limb at baseline examination, the limb that became lame after application of a diagnostic block on the limb with primary lameness, or the limb where localization of lameness [diagnostic blocks] was performed second after investigation of primary lameness) (Johnson et al., 2021); and compensatory lameness (after applying the diagnostic block on the primary limb, asymmetry intensity was reduced or eliminated on a limb at the opposite end of the body (Baxter et al., 2020), being considered in this work a reduction of at least 50% in asymmetry movements) (Figure 1).

### Lameness type

According to the software of the objective evaluation system, the lameness type was classified as impact, stance, and pushoff lameness for the forelimbs based on the Hdmax value and signals, and only pushoff (PDmax) and impact (PDmin) for the hindlimbs. Lameness intensity was based on the amplitude of asymmetry (mm) provided by the objective evaluation system used in this study. The VS value was used for the forelimbs, whereas the PDmax (pushoff lameness) and PDmin (impact lameness) values were used for the hindlimbs.

### Lameness intensity

The intensity was classified as mild, mild to moderate, moderate, moderate to severe, and severe.

### Lameness location

The lameness location was divided into five regions based on the response to diagnostic blocks and/or imaging examinations. One was the distal region (animals that responded positively to diagnostic blocks on the hoof: navicular bursa block, distal interphalangeal joint block, or palmar/plantar digital block) and the pastern (proximal interphalangeal joint block and abaxial sesamoid block). Another was the mid-region: animals responded positively to the diagnostic blocks of the metacarpal/metatarsophalangeal joint and the low four-point block. There was also the lower proximal region (animals that responded positively to the diagnostic block of the origin of the suspensory ligament in the forelimbs and the deep branch of the lateral plantar nerve in the hindlimbs). In addition, there was the mid-proximal region (animals that responded positively to diagnostic blocks of the radiocarpal and intercarpal joints in the forelimbs and tarsometatarsal and distal intertarsal joints in the hindlimbs). Finally, the upper proximal region corresponds to the animals that responded positively to diagnostic blocks of the femoro-tibio-patellar, medial femorotibial, and lateral femorotibial joints.

## Results

### Animals

**Figure 2:**
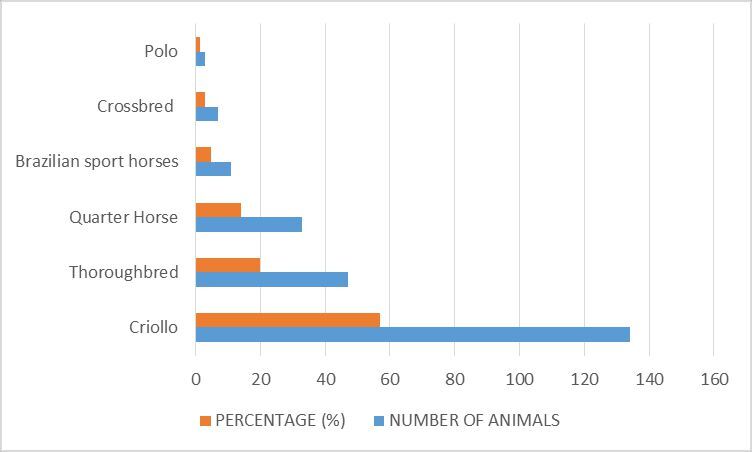
Number of animals and percentage relative to the breeds analyzed in the study.

In total, 235 animals were included in this study. The mean age of the animals was 7.1 years (range, 2–27 years), 47.7% were female (112 animals) and 52.3% male (123 animals). The most affected breeds are shown in Figure 2. The equestrian activities with the highest degree of representation were the *Freio de Ouro* competition, with 29,8% of the horses evaluated, followed by show jumping, with 17% of the horses, and mounted patrol police horses, with 10%. Other activities that were also present, albeit to a lesser extent, were long rope, morphological competition, racing, equine-assisted therapy, reproduction, polo, taming, walking, reining, *paleteada* competition, sale, pratical classes, and uninformed use.

### Presented lameness

Regarding lameness pattern distribution, 220/235 horses presented with lameness (93,6%), and only 15 horses did not have any kind of lameness (6,4%). Of these, 131/220 horses had lameness of the forelimb (59,5%) and 89 horses of the hindlimb (40,5%).

### Lameness condition

**Figure 3:**
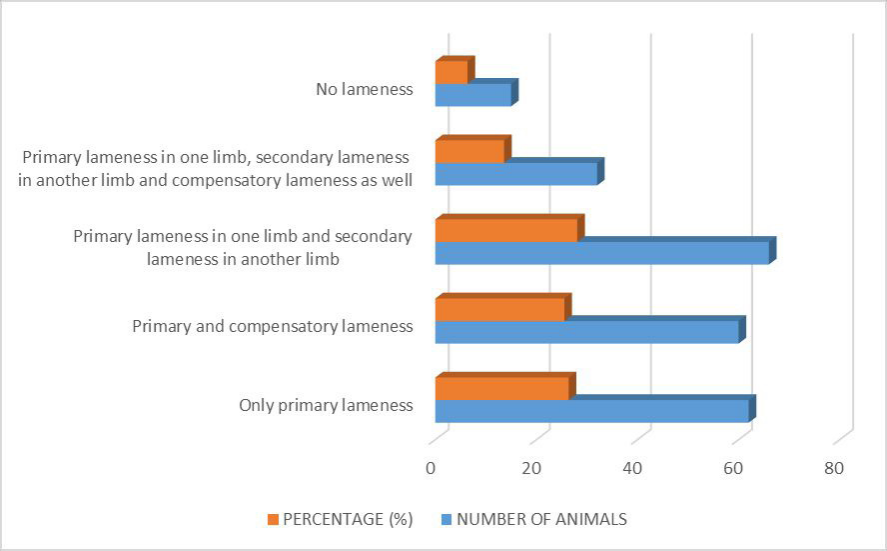
Number of animals and percentage according to lameness condition.

**Figure 4:**
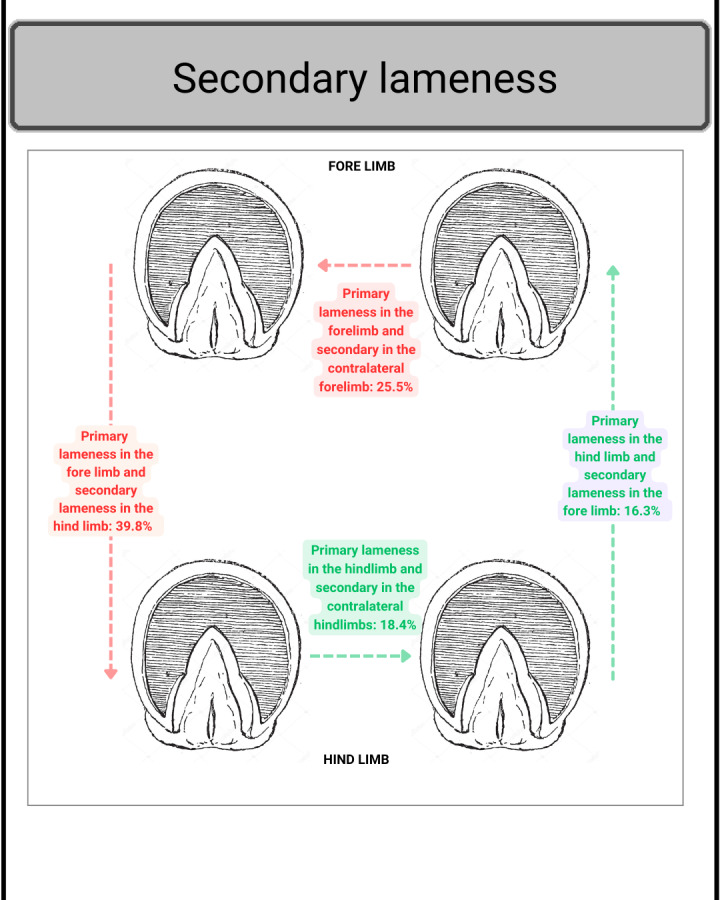
Primary and secondary lameness in the respective limbs.

The lameness conditions are shown in Figure 3. Considering the animals that presented with secondary lameness, Figure 4 shows the distribution of secondary lameness.

**Figure 5:**
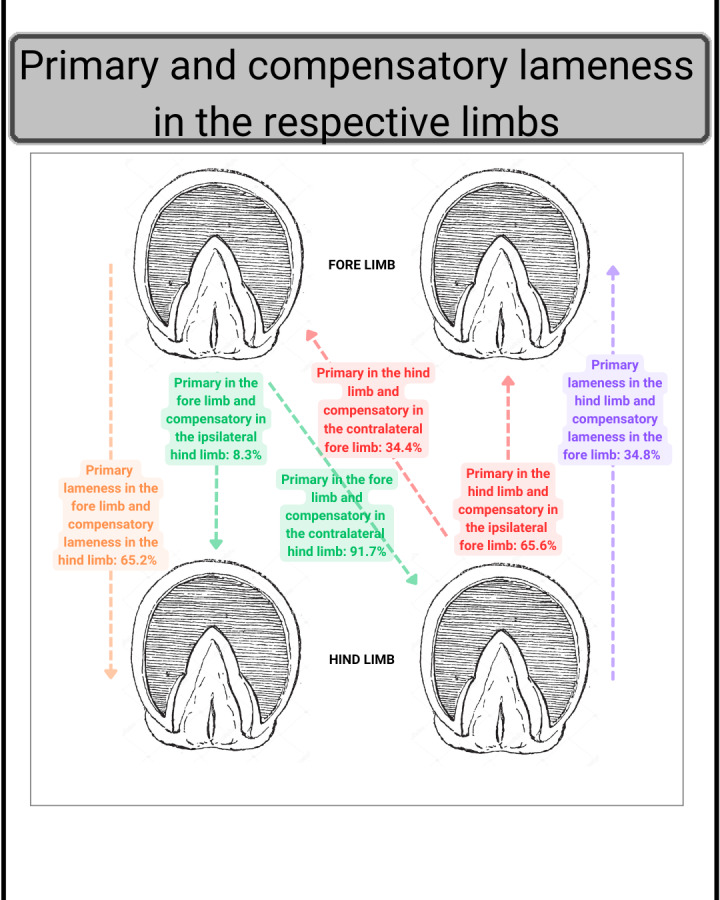
Primary and compensatory lameness in the respective limbs.

Regarding compensatory lameness, 65,2% of the horses had the condition in the hindlimbs because of primary lameness located in one of the forelimbs, while 34,8% of the animals had compensatory lameness in one of the forelimbs because of primary lameness in one of the hindlimbs. Figure 5 shows the distribution of compensatory lameness according to the limb with primary lameness and its most frequent compensatory lameness.

### Lameness type

**Figure 6:**
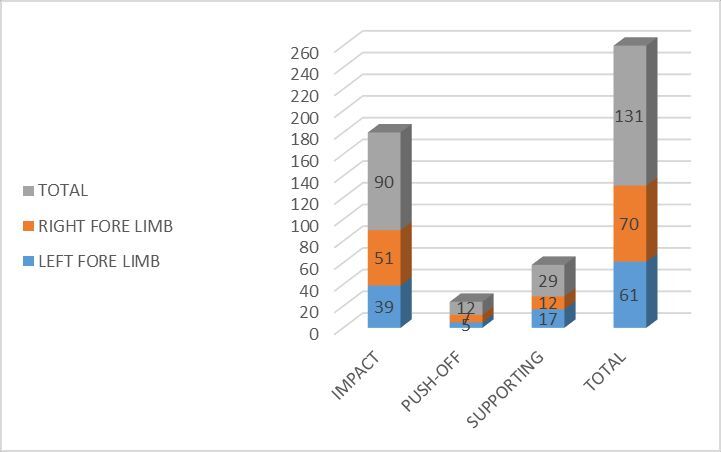
Frequency of forelimb lameness separated by the left and right limbs.

**Figure 7:**
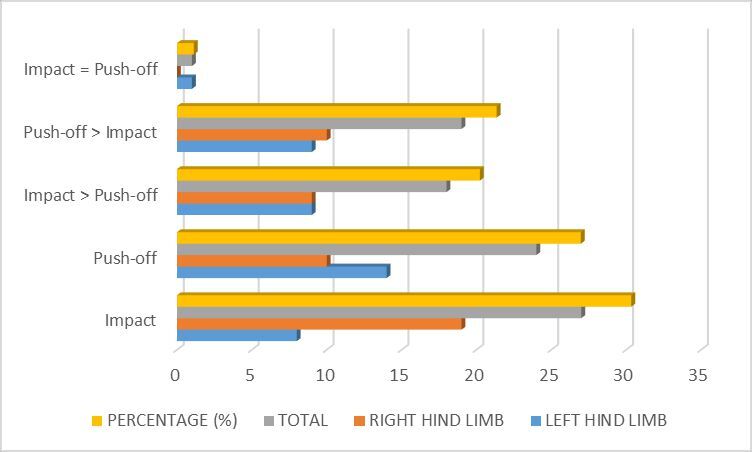
Type of lameness of hindlimbs separated by the left and right limbs.

The forelimbs presented with impact lameness in 68,7% of the cases, midstance lameness in 22,1%, and pushoff lameness in 9,2%. Regarding the hindlimbs, 30,3% had impact lameness, 27% had pushoff lameness, 21,3% had pushoff lameness greater than impact lameness, and 20% had impact lameness more evident than pushoff lameness. Figures 6 and 7 present the detailed data on the lameness type in relation to the affected limb.

### Lameness intensity

**Figure 8:**
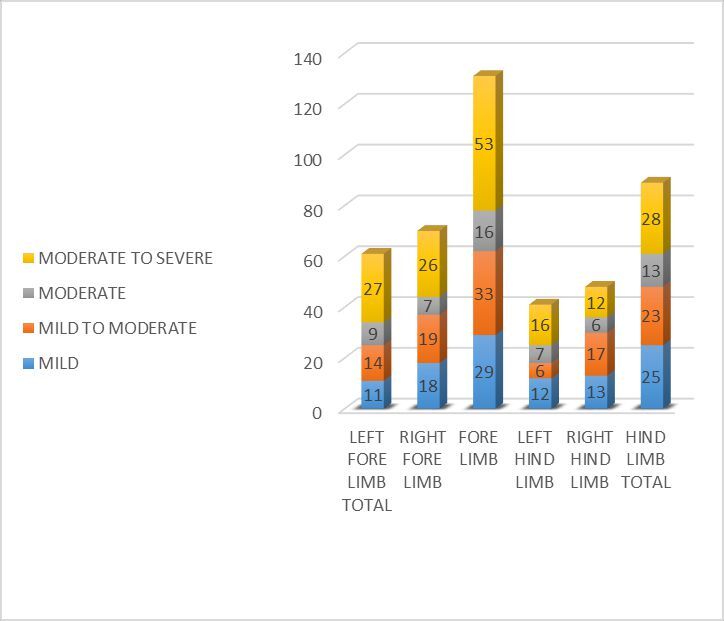
Lameness intensity in relation to presence in the fore- and hindlimbs. Expressed in numbers.

Lameness was mild in intensity in 24,5% of the horses, 25,5% had mild to moderate lameness, 13,2% had moderate lameness, and 36,8% had moderate to severe lameness. Figure 8 shows the lameness intensity for each limb.

### Lameness location

Considering the affected forelimb regions (Table 1), 79,4% of the horses presented with lameness in the distal region, 12,2% in the mid-region, and 8,4% in the lower proximal region. In the hindlimbs, 38,2% of the horses presented with lameness originating in the mid-proximal region, 22,5% in the lower proximal region, 19,1% in the mid region, and 18% in the upper proximal region. Table 1 provides a detailed presentation of the regions and the percentage of lameness per affected limb.

**Table 1:** Lameness location.

**LOCATION**	**DISTAL REGION**	**MID REGION**	**LOWER PROXIMAL REGION**	**MID PROXIMAL REGION**	**UPPER PROXIMAL REGION**
LEFT FORE LIMB	50	7	4	0	0
RIGHT FORE LIMB	54	9	7	0	0
**TOTAL**	**104**	**16**	**11**	**0**	**0**
**FORELIMBS PERCENTAGE (%)**	**79.4**	**12.2**	**8.4**	**0.0**	**0.0**
LEFT HINDLIMB		9	9	16	7
RIGHT HINDLIMB	2	8	11	18	9
**TOTAL**	**2**	**17**	**20**	**34**	**16**
**HINDLIMBS PERCENTAGE (%)**	**2.2**	**19.1**	**22.5**	**38.2**	**18.0**
**GRAND TOTAL**	**106**	**33**	**31**	**34**	**16**

## Discussion

The greater representation of Criollo breed horses, as well as the equestrian activity *Freio de Ouro*, can be explained by the geographical region where this study was conducted, southern Brazil. According to data from the Associação Brasileira de Criadores de Cavalos Crioulos (Tipa Júnior, 2020), 97% of the breed's stock is located in the southern region of Brazil.

The higher frequency of forelimb lameness than hindlimb lameness corroborates the findings of Dabareiner et al. (2005) and Mora-Carreno et al. (2014), who found a higher frequency of forelimb lameness in Chilean rodeo horses. This can be explained by the fact that the center of gravity, and consequently, the support of 60% of the horse's weight, is closer to the forelimb region (Beeman, 2008). In contrast, a study with Criollo horses reported 47,1% of lameness cases in the forelimbs and 52,9% in the hindlimbs (Abreu et al., 2011). Moreover, according to Swor et al. (2019), who studied Quarter and Paint Horses, the presence of lameness was higher in the hindlimbs than in the forelimbs. In these cases, the explanation may be based on the type of movement that the animals perform in their activities, where there is an overload and transfer of most of the horse's weight to the hindlimbs, which causes great stress and torque on the joints and other structures of the hindlimbs (Noble, 2001; Scott, 2008).

In the present study, the most frequent finding was primary lameness with secondary lameness, followed by primary lameness. These data are similar to those in the study by Johnson et al. (2021), in which another limb was also affected by secondary lameness in 56.3% of lameness cases. In contrast, a study on endurance horses showed that most animals had only one limb with lameness (Nagy et al., 2017). This difference appears to be related to the training method adopted, as endurance horses are not required to work in circles and tight turns, whereas horses training for other disciplines are. Training in a circle, whether ridden or not, may result in the exacerbation of lameness or identification of injury in more than one limb (Dyson & Greve, 2016; Nagy et al., 2017).

Considering the high proportion of lameness in more than one limb, it is important to identify the limb with primary lameness and the limb with compensatory lameness. In addition, evaluating the response to anesthetic blocks in horses with fore- and hindlimb lameness is useful for defining the effect of lameness in one limb on the movement of the other limbs (Maliye et al., 2015). Maliye et al. (2015) demonstrated that 50% of horses had primary lameness in the forelimbs and compensatory lameness in the contralateral hindlimb, while 21% had primary lameness in the forelimbs and compensatory lameness in the ipsilateral hindlimb. However, in our study, the values ​​ differed, with 91.7% of horses having primary lameness in the forelimbs and compensatory lameness in the contralateral hind limb, whereas 8.3% had primary lameness in the forelimbs and compensatory lameness in the ipsilateral hindlimb. In addition to the study by Maliye et al. (2015), Kelmer et al. (2005) reported the relationship between the induced changes in head movement and pelvic movement. However, the percentages of animals with primary lameness in the forelimb and compensatory lameness in the hindlimb were much higher in our clinical study, despite the standardized methods used in previous treadmill research (Kelmer et al., 2005). Animals with this compensatory lameness pattern support the second principle of the law of sides, in which forelimb lameness causes the horse to shift its center of gravity slightly toward the hind half of its body during the stance phase of the affected forelimb. This causes the pelvis to drop more on the contralateral hindlimb than on the ipsilateral hindlimb and also causes the pelvis to rise less when the horse moves forward (Kelmer et al., 2005; Maliye et al., 2015; Maliye & Marshall, 2016).

The other animals in our study had primary hindlimb lameness and compensatory lameness in the forelimbs. Of these, 65,6% had compensatory lameness in the ipsilateral forelimb. However, Maliye and Marshall (2016) reported different results for this type of compensatory lameness, with a percentage of 27% of horses. These findings corroborate the first principle of the law of sides, whereby during hindlimb stance, the horse transfers weight forward onto the contralateral forelimb, which simultaneously supports the weight; thus, the head drops more than normal. Therefore, the normal head drop during weight support of the opposite forelimb is less, resulting in the appearance of lameness of the ipsilateral forelimb (Kelmer et al., 2005; Maliye et al., 2015; Maliye & Marshall, 2016).

According to Maliye and Marshall (2016), evaluation of the compensatory effect of lameness enabled them to determine that hindlimb lameness can have load redistribution and a compensatory effect on the ipsilateral forelimb, but hindlimb lameness did not reflect compensatory lameness in the contralateral forelimbs. However, in our study, a considerable percentage of the horses had primary lameness in one hindlimb, with compensatory lameness in the contralateral forelimb. In a study by Reed et al. (2020), in the 15,6% of horses that demonstrated forelimb lameness on assessment with the wireless inertial sensor system, the primary abnormality was found in the hindlimb, and in 18,4% that demonstrated hindlimb lameness with the wireless inertial sensor system, a primary abnormality was found in the forelimb, which suggested compensatory lameness with primary lameness below the detection threshold.

Among the animals with secondary lameness, the majority had primary lameness in one forelimb and secondary lameness in the hindlimb, followed by primary lameness in the forelimbs and secondary lameness in the contralateral forelimb. These results differ from those reported by Johnson et al. (2021), who found a higher percentage of animals with secondary lameness in the forelimbs (57,1%) than in the hindlimbs (13,2%). Secondary lameness usually occurs on the opposite side of the primary lameness and is potentially caused by chronic weight-bearing overload that causes pain in the entire limb or in the supporting structures of the contralateral limb (Keegan, 2022).

Impact lameness was more frequent in both the fore- and hindlimbs, followed by midstance in the forelimb and pushoff in the hindlimb. Azevedo et al. (2015) examined horses on three surface types (concrete, sand, and grass) and found a higher frequency of animals with impact lameness in both limbs. The higher impact lameness can be explained by the floor used for examining the animals, which was mostly hard.

Regarding the lameness intensity, most of the horses in this study presented moderate-to-severe lameness, followed by mild-to-moderate and then moderate lameness. Johnson et al. (2021), using the American Association of Equine Practitioners (AAEP) scale (0-5), reported a mean grade of lameness of 2.34 for forelimbs and 2.21 for hindlimbs. Broster et al. (2009), on the other hand, found that 87% of the horses in their study had at least one limb with a lameness grade of 3 or 4 (0-4), but when they considered the limbs separately, 94% had a lameness grade of 2 or 3. Other studies using similar lameness assessment scales found animals with lower lameness intensities. For example, Mora-Carreno et al. (2014) found that 43% of the animals had grade 2 lameness and 28% had grade 1 lameness.

In this study, the distal region of the forelimb was the most common source of lameness, followed by the mid-(metacarpophalangeal joint) and lower proximal (proximal metacarpal) regions. These data are similar to those reported by Tank et al. (2020), with a higher percentage of lesions affecting the hoof, followed by the metacarpophalangeal joint. Abreu et al. (2011) evaluated Criollo horses, the most common breed in our study and found a high percentage of animals with lameness in the distal region of the forelimb. The high prevalence of distal limb changes may be related to poor hoof trimming and shoeing (Naeini & Niak, 2005), which predisposes to pain in the hoof (Scott, 2008). This is in agreement with Canto et al. (2006), who identified a high frequency of hoof changes in Criollo horses during training.

In the hindlimbs, the most frequently affected region was the mid-proximal region, followed by the lower proximal, mid-, upper-proximal, and distal regions. Similarly, other studies have identified the tarsal region as the main origin of hindlimb lameness, as observed in dressage horses (Murray et al., 2010), athletic Criollo horses (Abreu et al., 2011) and Western competitive animals (Johnson et al., 2021). A high number of injuries in the tarsal region, especially the distal intertarsal and tarsometatarsal joints, occur because of the transfer of most of the horse's weight to the hindlimbs during maneuvers, which involve rapid changes in direction and abrupt stops. Thus, a large amount of tension and torque are applied to these joints (Noble, 2001; Scott, 2008). Similar to what was found in this study and in the study by Abreu et al. (2011), most of the horses were Criollo horses that participated in the *Freio de Ouro* competition, which is a type of Western competition in which the animals are required to bump, turn, and make sudden changes in direction in some stages of the race, as well as other repetitive movements that end up overloading certain anatomical structures of the hindlimb.

The main limitation of this study is the extremely high number of animals with lameness. However, these data may be overestimated, given that they were from a retrospective study in which the group of evaluated animals included animals already suspected of lameness. In addition, the number of animals can be considered low compared with those in other studies with similar objectives. Another limitation we deem important is that the results were mainly based on the response to diagnostic blocks, determining the region and not the specific anatomical structures. Although the diagnostic blocks were performed in a standardized manner, starting distally and proceeding proximally as needed, it is important to emphasize the potential for local diffusion and consequent damage that can occur with the specificity of the diagnostic blocks.

Future retrospective studies should evaluate horses referred for lameness examinations, and include animals considered healthy by their owners. It is also important to specifically diagnose the origin of lameness using complementary examinations to ensure greater accuracy.

## Conclusions

Lameness primarily affects the forelimbs, with primary forelimb lameness and secondary hindlimb lameness being the most common condition. Compensatory lameness often occurs in the hindlimb opposite to the affected forelimb. Impact lameness is the most common primary lameness, typically of moderate to severe intensity, in both the fore- and hindlimbs. Forelimb lameness is mostly distal (pastern and hoof), whereas hindlimb lameness is most frequent in the mid-proximal region (tarsus).
